# SIMPL: A Simplified Model-Based Program for the Analysis and Visualization of Groundwater Rebound in Abandoned Mines to Prevent Contamination of Water and Soils by Acid Mine Drainage

**DOI:** 10.3390/ijerph15050951

**Published:** 2018-05-10

**Authors:** Sung-Min Kim, Yosoon Choi

**Affiliations:** 1 Division of Graduate Education for Sustainability of Foundation Energy, Seoul National University, Seoul 08826, Korea; snuhyrule@hanmail.net; 2 Department of Energy Resources Engineering, Pukyong National University, Busan 48513, Korea

**Keywords:** acid mine drainage, abandoned mines, groundwater rebound, Dongwon coal mine, Dalsung copper mine

## Abstract

Cessation of dewatering following underground mine closure typically results in groundwater rebound, because mine voids and surrounding strata undergo flooding up to the levels of the decant points, such as shafts and drifts. SIMPL (Simplified groundwater program In Mine workings using the Pipe equation and Lumped parameter model), a simplified lumped parameter model-based program for predicting groundwater levels in abandoned mines, is presented herein. The program comprises a simulation engine module, 3D visualization module, and graphical user interface, which aids data processing, analysis, and visualization of results. The 3D viewer facilitates effective visualization of the predicted groundwater level rebound phenomenon together with a topographic map, mine drift, goaf, and geological properties from borehole data. SIMPL is applied to data from the Dongwon coal mine and Dalsung copper mine in Korea, with strong similarities in simulated and observed results. By considering mine workings and interpond connections, SIMPL can thus be used to effectively analyze and visualize groundwater rebound. In addition, the predictions by SIMPL can be utilized to prevent the surrounding environment (water and soil) from being polluted by acid mine drainage.

## 1. Introduction

In operational mines, groundwater flooding of the working area is prevented by continuous pumping operations. However, once this pumping ceases, when a mine is abandoned, the groundwater level in the mine begins to rise again—a phenomenon called “groundwater rebound.” When this phenomenon occurs, groundwater either gradually flows back into the underground voids that were generated, due to mining activities or flows back into the strata near the mine. As a result, the groundwater level rises up to the groundwater discharge point; this is the level above which groundwater flowing into the mine flows back out to the surface or into surrounding aquifers, and is typically associated with mines because of artificial structures, such as shafts or drifts, built for mining activities.

Predicting the groundwater rebound phenomenon in a mine area is important for two reasons. First, a plan for draining the groundwater flowing into the pit can be established based on this prediction. Second, in an abandoned mine, this prediction can be utilized to prevent the surrounding environment (water and soil) from being polluted by acid mine drainage. There have been many studies on the assessment and prevention of environmental pollution due to acid mine drainage [1,2,3]. In particular, through prediction of the groundwater rebound phenomenon, we can forecast when groundwater that has flowed into a mining void after mine abandonment will fill the void and flow out and where this flow will occur. The combination of these predictions with mine drainage quality prediction technology enables the comprehensive assessment of changes in water quality resulting from mine outflow [4].

The quantitative information provided by mine groundwater rebound prediction technology makes it one of several important technologies for preventing mine-associated damage in areas with abandoned mines. Many case studies have been reported that consider the development and field application of models for groundwater rebound prediction. Toran and Bradbury [5] analyzed groundwater rebound in the vicinity of an abandoned lead and zinc mine using the MODFLOW model (United States Geological Survey, Virginia, United States), a representative finite difference groundwater flow model based on the Darcian groundwater flow equation [6]. Similarly, Sherwood [7] applied the MODFLOW model to predict the groundwater rebound associated with a large-scale coal field in the United Kingdom. Huisamen and Wolkersdorfer [8] estimated the hydrogeochemical evolution of mine water over time, based on MODFLOW model using groundwater monitoring data and geochemical analyses. However, as the groundwater environment in mines is physically and hydraulically different from a “typical” groundwater environment, analysis of the mine groundwater rebound phenomenon using a conventional groundwater flow model, such as MODFLOW, presents many challenges [4]. In particular, artificial structures built for mining activities, such as mine voids, shafts, and drifts, may have a significant effect on the hydraulic environment of groundwater in the mine. Although the flow of groundwater in the strata surrounding a mine void can be assumed to be a laminar groundwater flow, flow in the mine void itself is turbulent. Accordingly, for prediction of the mine groundwater rebound phenomenon, it is not appropriate to use existing groundwater flow models based on the conventional Darcian groundwater flow equation [9], which assumes laminar flow; an alternative prediction method is required that accounts for the hydraulic characteristics specific to the mines.

To overcome the limitations of the MODFLOW model, MINEDW (Itasca Denver, Inc., Colorado, United States), a three-dimensional finite element groundwater flow code specifically for mining applications was developed [10], based on the algorithms of Durbin and Berenbrock [11]. It was designed to quantify the more detailed problems of the configuration of the phreatic surface and inflows to underground openings by supplementing inadequate discretization, poor representation of seepage faces, and the hydrodynamics of flow at discrete discharge points. A finite element grid facilitates the representation of complex geometries and highly-variable spatial discretization, which is particularly useful for mining applications with complex geologic structures and steep hydraulic gradients. In addition, MINEDW accounts for local resistance to flow at relatively constricted discharge points, such as drifts or drainholes, more effectively. However, although MINEDW has been specially designed to address the relatively unique hydrologic complexities of mining, it has limitations with respect to covering the relatively small portion of the region and requiring detailed data of the study area.

Younger and Adams [12] made predictions of the groundwater rebound phenomenon for a coal field in Whittle and Shilbottle, UK. In this study, since it was possible to use detailed underground maps and groundwater level measurement data across many locations, changes in the groundwater level and surface runoff could be predicted at 2 h intervals using the physically based, fully 3D VSS-NET model of SHETRAN (Newcastle University, Newcastle upon Tyne, United Kingdom). In addition, Adams and Younger [13] utilized the VSS-NET model to predict the groundwater rebound phenomenon in a tin mine in South Crofty, UK. However, although the VSS-NET model can perform precise analyses for small areas, its large data input requirements mean that the simulation efficiency drops sharply as the spatial scale increases.

The GRAM model, a semi-distributed model, was developed by Sherwood [7] by simplifying the remaining mine area: the connection structure between the spatially distributed mine workings in the fully 3D groundwater flow model is excluded. The GRAM model (Newcastle University, Newcastle upon Tyne, United Kingdom) is a lumped parameter model that treats mine areas as interconnected ponds and pipes. To model the water flow in a pond or the outflow of water to the surface, the intra-pipe fluid flow equation is used. Several studies using the GRAM model have been carried out to analyze the mine groundwater rebound phenomenon associated with a coal field in South Yorkshire, UK [14,15,16]. Although the GRAM model simplifies the underground environments as ponds and pipes, it still requires several parameters that are hard to acquire in abandoned mines. In addition, it only considers ponds to be interconnected with a horizontal pipe, of which both ends are at the same height. Recently, Choi et al. [17,18] developed a Windows console-type program using the FORTRAN language (International Business Machines Corporation (IBM), New York, United States) that facilitates the application of the GRAM model to abandoned mine areas. However, no such program has been developed with a Graphical User Interface (GUI); a GUI would simplify data processing and enable effective visualization of the analytical results. As a result, the GRAM model has been only rarely utilized for practical applications such as mine reclamation programs. Recently, a neural network model has been used to predict the groundwater rebound process at a restored open cut coal site [19], and automated electrical resistivity tomography was considered as a means of monitoring groundwater rebound in an operational mine site [20].

Since the possibility of data collection in abandoned mine sites is generally limited, the data input requirements of these models can be a disadvantage for field applications. The objective of this paper is to facilitate predictions of the groundwater rebound phenomena in abandoned mine areas by developing a new, user-friendly, simple program in which results can be visualized as intuitive 3D models. The results of the groundwater rebound represented by a 3D model can help researchers intuitively analyze the possibility of acid mine drainage generation in abandoned mines. We propose a new program, SIMPL (Simplified groundwater program In Mine workings using the Pipe equation and Lumped parameter model), by using a simplified model that demands very few parameters for predicting groundwater rebound in abandoned mines. It uses the only standard pipe equation, that does not need iteration to solve, for the direct modeling of the flow processes. The hydraulic conductivity of a pond is assumed to be sufficiently large in the model that the hydraulic gradient of the pond is ignorable. Therefore, there is no problem of acquiring various data, which arises when trying to apply traditional groundwater models to groundwater rebound. We also present results of a case study in the Dongwon coal mine and the Dalsung copper mine in the Republic of Korea, using our new SIMPL program to model groundwater levels at a range of spatial and temporal scales.

## 2. Principles of the Simplified Model

**Figure 1:**
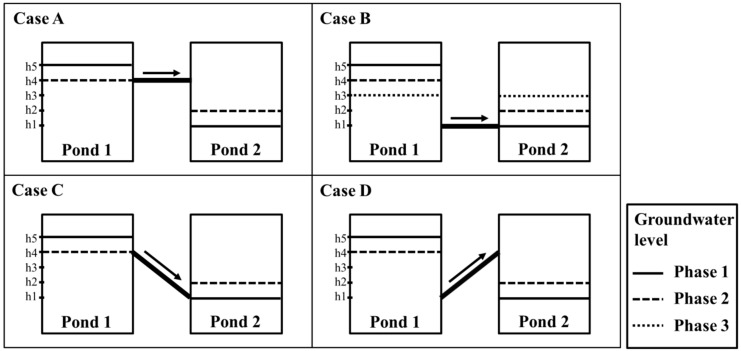
Schematic diagram of the simplified model in SIMPL (Simplified groundwater program In Mine workings using the Pipe equation and Lumped parameter model) showing the groundwater flow through each pipe type.

**Figure 2:**
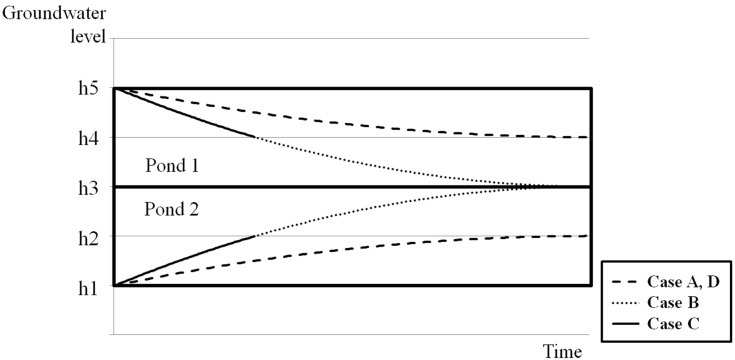
Groundwater level variations according to time for each case in Figure 1.

A simplified model, abstracting mine areas in the forms of ponds and pipes connected to each other, is used in this study. In this model, a pond represents a mine working surrounded by impermeable layers, and a pipe represents the discrete overflow points connecting the mine workings. For the mine workings, diverse geometric forms can be considered. Typical features that form interpond overflow points include drifts, boreholes, and permeable geological features, such as dykes, limestone beds, or an open fault. The conceptualization method of SIMPL for underground environments is similar to that of the GRAM model. A schematic diagram of the simplified model used in SIMPL is shown in Figure 1. Unlike the GRAM model, which only considers a horizontal pipe, as in cases A and B in Figure 1, SIMPL can additionally consider an inclined pipe, as in case C or D. Sometimes, a horizontal pipe cannot sufficiently explain the groundwater flow in abandoned mine workings. Access to deep mines is achieved through tunnels varying in inclination from horizontal to vertical, and a decline means that an inclined tunnel can be a pipe connecting ponds. In addition, permeable geological features, like an inclined fault plane, can also perform the role of the connecting pipe between ponds. Figure 2 shows the groundwater level variations according to time for each case in Figure 1. In these cases, the conditions of Ponds 1 and 2, such as the storage coefficient, pond area, and precipitation, are supposed to be equal. In addition, any inflow or outflow, except for the flow through the connecting pipe, was not considered. The pipe is fully flooded when groundwater is flowing along a pipe. The velocity of the flow in the pipe is determined by the hydraulic head difference between the ponds, according to the pipe flow equation. Because the hydraulic head difference between the two ponds is the same in cases A and D, both groundwater level variations are equal. The groundwater level of Pond 1 decreases until it reaches the height of the upper end of the pipe. Although the groundwater level variations in cases A and D are equal in this example, there can be a notable distinction with different conditions in the pond and pipe. The hydraulic head difference in case B or C is larger than that of case A or D. Therefore, the velocity of case B or C is higher than that of case A or D, and the groundwater level of Pond 1 decreases more rapidly in cases B and C. In case B, the groundwater level of Pond 1 decreases until it becomes equal to the level of Pond 2. On the other hand, in case C, the level of Pond 1 decreases until it reaches the height of the upper end of the pipe.

SIMPL predicts a mine groundwater rebound phenomenon according to the five-step calculation procedure using the pipe equation and lumped parameter. First, the amount of rainwater that flows into each pond representing a mine working is calculated. The amount of rainwater recharged into each pond is calculated by accounting for the rainfall that reaches the catchment area of each pond. The total rainfall during the period of interest is first determined, and then the effective rainfall is calculated by subtracting the evaporation from the amount of rainwater that reaches the earth’s surface. If the amount of evaporation is larger than the amount of rainfall, the effective rainfall is calculated to be zero. The amount of recharged rainwater is obtained by subtracting the surface runoff from the total effective rainfall. Second, if pumping work is in progress in the mine, the amount of groundwater that is discharged to the outside environment from each pond is calculated and deducted. Third, if groundwater flows into the mine from the sea, adjacent mines, or adjacent aquifers, the amount of groundwater that flows into each pond is calculated and added. Once the amount of groundwater existing in each pond is calculated up to the third step, the fourth step is then calculated by determining the amount of groundwater moving between the connected ponds using the pipe flow equation. Finally, the groundwater level in each pond is calculated by taking into account the amount of groundwater that has been transferred. If the groundwater level in a pond rises and overflows, in the fifth step, the amount of groundwater that flows out onto the earth’s surface is calculated using the pipe flow equation.

In the simplified model, the storage coefficient is determined through the calibration process of the input variables using the water level measurement data of the pond. Accordingly, to calibrate the storage coefficient, the existing measurement data for the water level are required. Especially in SIMPL, the hydraulic characteristics of a pond can be considered to be heterogeneous in the vertical direction. For example, if a roof collapse occurs as a result of mining works fully filling the goaf with cataclastic rocks, the storage coefficient may be much higher than that of the surrounding strata. To reflect such heterogeneity in the vertical direction, a pond is divided into multiple layers in the vertical direction in SIMPL. The calculation from the first step to the fifth step is repeatedly carried out for each pond for the predetermined simulation period.

The simplified model calculates the head loss in each pond connected with a pipe to model the movement of groundwater between ponds and the outflow of mine drainage to the land surface (Equation (1)). If groundwater is flowing along a pipe, the pipe must be fully flooded, since the height of the location at which the pipe is connected must be lower than the groundwater level in at least one of the connected ponds. The total head loss of the pond in Equation (1) is calculated by adding the friction head loss and the minor head losses, which occur during inflow and outflow. The friction head loss is calculated using the Darcy–Weisbach equation [21]:

(1) $$∆H=\frac{0.5V^{2}}{2g}+\frac{V^{2}}{2g}+\frac{{\lambda \mathrm{LV}}^{2}}{2\mathrm{gD}},$$

where ∆H is the total head loss (m), V is the flow velocity of groundwater in the pipe (m/s), λ is the coefficient of friction loss determined by the surface roughness and diameter of the pipe, L is the length (m) of the pipe, g is the acceleration of gravity, and D is the diameter (m) of the pipe. Each term in Equation (1) reflects the following: $0.5V^{2}/2g$ is the head loss that occurs when the pond groundwater flows into the pipe (inlet loss); $V^{2}/2g$ is the head loss that occurs when the groundwater flows out of the pipe (outlet loss); and ${\lambda \mathrm{LV}}^{2}/2\mathrm{gD}$ represents the friction head loss that occurs in the pipe.

Equation (1) can be rearranged using the following expression to make velocity

(2) $$V=\sqrt{\frac{2g∆H}{\left(1.5+\frac{\lambda L}{D}\right)}}.$$

If fluid flow is turbulent, the value of λ can be determined using the Prandtl–Nikuradse or Colebrook–White equation. The Prandtl–Nikuradse equation assumes turbulent fluid flow [21]. For a turbulent flow, the value of λ is expressed as a function of roughness relative to pipe diameter (k/D):

(3) $$\lambda ={\left(\frac{1}{2\log\frac{3.7D}{k}}\right)}^{2},$$

where k is the roughness coefficient (m) of the pipe surface. The Prandtl–Nikuradse equation does not require an iterative process to obtain the solution; the code used to solve the Prandtl–Nikuradse equation is thus very simple, and the calculation time is very short. However, this equation cannot be applied if the fluid flow is not turbulent.

The Colebrook–White equation is commonly used to calculate the turbulent fluid flow in a pipe. The Colebrook–White equation calculates the value of λ by taking into account both rough and smooth turbulent flows:

(4) $$\frac{1}{\sqrt{\lambda }}=-2\log\left[\frac{k}{3.7D}+\frac{2.51\nu }{\mathrm{VD}\sqrt{\lambda }}\right],$$

where ν is the kinematic viscosity (m^2^/s) of the groundwater, which is the ratio of the viscosity (μ) to the unit weight (ρ) of the fluid. In the temperature and pressure conditions typical of most groundwater environments, changes in the kinematic viscosity are very small. Accordingly, the kinematic viscosity can be considered a constant. Freeze and Cherry [22] reported that the kinematic viscosity of fluid was 1.124 × 10^−6^ (m^2^/s) for a temperature of 15.5 °C, density of 1000 kg/m^3^, and viscosity of 1.146 × 10^−4^ kgf·s/m^2^.

To calculate the flow velocity in the pipe, the GRAM model uses either the Prandtl–Nikuradse equation or the Colebrook–White equation. However, although the codes used to solve the equations do not require many calculations, at least three parameters, like the length (L), diameter (D), and roughness coefficient (k) of the pipe, are required in the GRAM model. These parameters should be supposed before predicting the water level, and be modified after the calibration process to optimize them. Because of the difficulties in collecting data in an abandoned mine, it is very hard to define specific pipe characteristics representing the real connection between ponds. Therefore, it can be a wasteful task to modify each of the three parameters (L, D, k) to create an appropriate prediction in the GRAM model. In SIMPL, the parameters are more simplified as one parameter that unifies the other parameters. All parameters in Equation (2), except for the hydraulic head difference (∆H), were integrated into a parameter P based on the Prandtl–Nikuradse equation (Equation (3)). The velocity (Equation (2)) can be expressed as the following equation:

(5) $$V=P\sqrt{∆H}\;,\;P=\sqrt{\frac{2g}{\left(1.5+\frac{\lambda L}{D}\right)}}.$$

Because λ is determined only by D and k in the Prandtl–Nikuradse equation, P represents a constant that incorporates the characteristics of the pipe. Therefore, the calibration process can be simplified and automated in SIMPL using P rather than by using the method of the GRAM model. The SIMPL program also provides another prediction option using the exponential Hazen–Williams formula:

(6) $$V=0.35327\cdot C\cdot D^{0.63}{\left[\frac{∆H}{L}\right]}^{0.54},$$

where C is the Hazen–Williams flow coefficient for a pipe. Because C, D, and k are constant, the parameters of Equation (6), except for $∆H^{0.54}$, can also be simplified into one parameter for the iterative calculations in the calibration process.

## 3. SIMPL Program

### 3.1. SIMPL Program Interface

**Figure 3:**
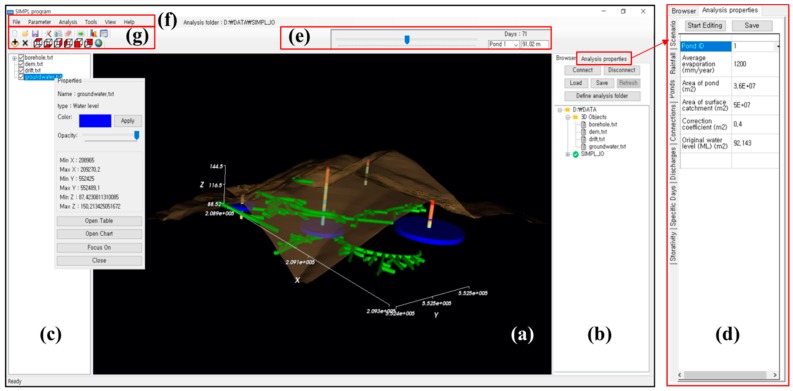
Graphical user interface of the SIMPL program. See the main text for label descriptions.

A new program, “SIMPL”, was developed using Visual Basic 2013 (Microsoft Corporation, Washington, United States) to predict and visualize the groundwater rebound phenomenon in an abandoned mine area. Figure 3 shows the main interface of the program, which comprises (a) a 3D viewer, (b) a file browser, (c) a 3D data tree window, (d) an analysis properties window, (e) a time step slider, (f) a menu bar, and (g) a toolbar.

**Figure 4:**
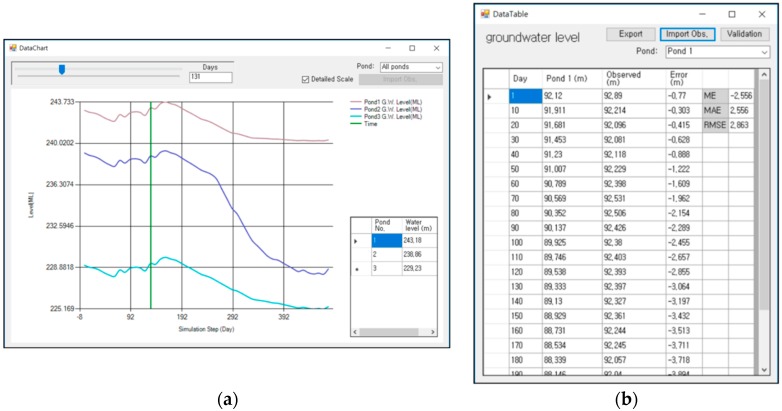
SIMPL program (**a**) chart tool and (**b**) table tool.

The 3D viewer was developed using the Visualization Toolkit (VTK, Kitware, NY, USA) which is an open-source, freely available software system for 3D computer graphics, image processing, and visualization. To enable effective visualization of the predicted groundwater level and rebound phenomenon, the SIMPL program can display a topographic map, mine drift, goaf (the part of the mine from which minerals have been removed), and geological properties from borehole data together with the predicted groundwater level. The file browser enables 3D mine data to be imported easily from the user’s computer; after entering the correct folder, the relevant file can be dragged into the 3D viewer for visualization. In addition, a target folder for both input and output data can be set in the file browser, and users can always return to a specific folder by saving the file browser settings. 3D objects displayed in the 3D viewer can be managed in the 3D data tree window: this window controls the display properties of each object, such as the color, opacity, visibility, and focus of the 3D viewer. The analysis properties window shows the parameters for the groundwater rebound simulation; these are input manually or imported from data input in the ASCII file format. These parameters can be edited in this window; changes to these parameters influence the simulation result. The SIMPL program shows the predicted groundwater level as a time-dependent 3D object by using the time step slider. In the menu bar and toolbar, modules such as the chart tool and table tool are presented for easy analysis and visualization. The chart tool (Figure 4a) can be used to immediately graph the results of the groundwater level prediction. In addition, the predicted result can be compared to observed data by importing observational data in the correct data format. To acquire exact values for the predicted groundwater level at each time step, the table tool (Figure 4b) can be used. The results can be exported into the CSV file format, which is readable by many other software packages. This tool also provides a function that validates the prediction results by comparing the results with observed data based on the root mean square error (RMSE).

### 3.2. SIMPL Program Simulation Engine Module

The input data required for SIMPL and the output data generated by the simulation are shown in Table 1. Data are input to the SIMPL program as a series of American Standard Code for Information Interchange (ASCII) files. Input data can be easily modified in basic text-editing software or in the analysis properties window of the SIMPL program.

**Figure 5:**
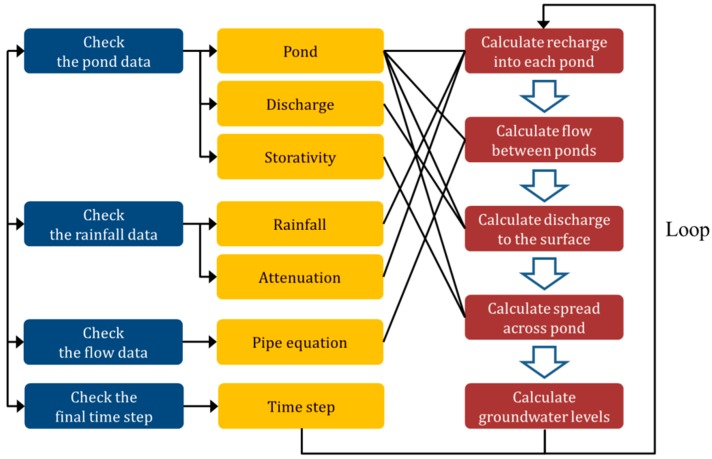
Flowchart showing the SIMPL program simulation engine module procedure.

SIMPL predicts the mine groundwater rebound phenomenon from these input data following the procedure in Figure 5, which is implemented by the simulation engine module in SIMPL. First, basic information about the conceptual model, such as the number of ponds, number of pond connections, number of rainfall data points, and number of storage data points should be input. After checking this, the amount of rainwater that flows into each pond representing a mine is calculated. This calculation first determines the rainfall that reaches the catchment area of each pond by determining the total rainfall during the period of interest; the total effective rainfall, i.e., the amount of rainwater that reaches the earth’s surface minus that which evaporates, is then calculated; finally, the amount of recharged rainwater is determined by subtracting surface runoff from total effective rainfall.

Once the amount of groundwater in each pond has been calculated, the amount of groundwater moving between the connected ponds is then calculated using the pipe flow equation. By considering the height and a parameter P that incorporates the roughness, diameter, and length of the pipes, the extent of the groundwater flow is determined. In the same way, the amount of groundwater that outflows to the discharge points is calculated by applying the pipe flow equation to the discharge data.

Finally, the groundwater level in each pond is calculated, taking into account the amount of groundwater that has been transported. The storage coefficient is determined in the simplified model through the process of calibration of the input variables using pond water level measurement data. Existing water level measurement data are thus required to calibrate the storage coefficient. To account for vertical heterogeneity, ponds are divided into multiple depth-determined layers. Consequently, as the groundwater level rises and reaches a new zone, the storage coefficient changes accordingly. The calculation is carried out repeatedly for each pond for the predetermined simulation period, and the result calculated in each loop is stored as the output data.

The input data required by the SIMPL program are relatively simple compared to those required by other groundwater flow models. Additional information about the area to be modeled, such as the hydraulic conductivity and porosity, is therefore not required by SIMPL, in contrast to conventional groundwater flow models.

### 3.3. 3D Visualization Module of the SIMPL Program

**Figure 6:**
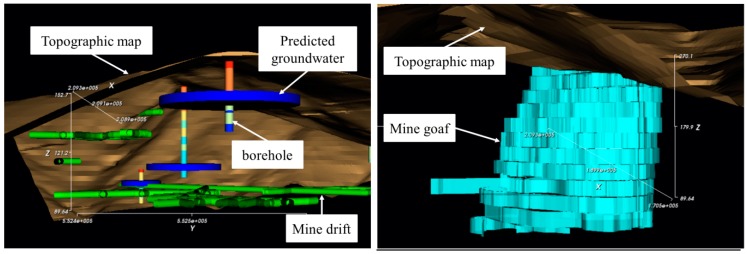
Examples of different 3D object data files.

**Figure 7:**
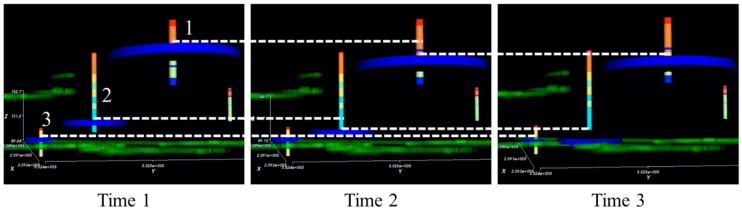
3D visualization of groundwater change by using the time step slider in the SIMPL program.

Groundwater level prediction results can be stored as 3D objects and visualized with other mine-related 3D objects in the SIMPL program; Table 2 shows the types and data structures of 3D objects in SIMPL. The 3D visualization module of the SIMPL program was coded in Visual Basic 2013 using VTK. First, the module reads the 3D object type from the first line of the TXT file format data file and determines the appropriate 3D modeling method for that object. The second and third lines of this data file are used differently, depending on the object type. From the fourth line, the *x*, *y*, and *z* coordinates of the points should always be entered in the same way, although there are some formatting differences among the types, which are outlined below. To model the topographic map, the vtkDelaunay2D class is utilized to create Delaunay triangles from the points with *x*, *y*, and *z* coordinates. To model the mine drifts and shafts, the vtkLineSource and vtkTubeFilter classes are utilized. The vtkLineSource class is used to determine the center line of the mine drift, and the vtkTubeFilter is used to create the tube along this center line. In this type of data file, each line in the data file records the *x*, *y*, and *z* coordinates of the start and end points of a center line. If the next center line of a tube is connected to the previous center line, the coordinates of the new start point should coincide with the coordinates of the previous end point. These VTK classes were utilized to model the borehole data. The label and class of the borehole data were also included in the file to subdivide measurement data by depth and value. Mine goaf is modeled based on the mine map of each level. Accurate determination of goaf shape is very difficult in most abandoned mine areas, so the SIMPL program models the shape of the goaf as polygons with the vtkPolygon class based on each level of the mine map, extruding the polygon to account for the vertical interval between the levels using the vtkLinearExtrusionFilter class. The groundwater level object is stored in each line of the file corresponding to the appropriate time step. In the SIMPL program, the groundwater level of each pond is modeled as a cylinder using the vtkCylinderSource class. Examples of each of these five file types are presented in Figure 6. By using the time step slider, the predicted groundwater level can be shown effectively as a time-dependent 3D object in the SIMPL program. Figure 7 shows the example of groundwater level changes over time. From time 1 to 2, the groundwater levels of Ponds 1 and 2 decrease evidently. From time 2 to 3, on the other hand, the groundwater levels of Ponds 2 and 3 (but not Pond 1) decrease evidently until they reach the mine drifts.

## 4. Application of SIMPL in Abandoned Mines

### 4.1. Dongwon Coal Mine

The Dongwon coal mine is located in the Gohan/Sabuk district of Gangwon-do, Republic of Korea, and produced approximately 2 million tons of coal between its opening in December 1963 (as the Dongwon Coal Field Development Co., Ltd., Busan Metropolitan City, Korea) until cessation of operations in 2004. The slant chute block caving coal mining method was the primary mining method used, with a vertical interval of 50 m between the levels. Mining work was carried out from 650 m above mean sea level (MSL), at the earth’s surface (0 level), and down to 50 m above MSL (13th level). During the period the mine was in operation, groundwater was removed at an average rate of 5250 m^3^/d to enable the mine to function; after the mine was abandoned, the groundwater level started to rise again once pumping stopped in February 2005.

**Figure 8:**
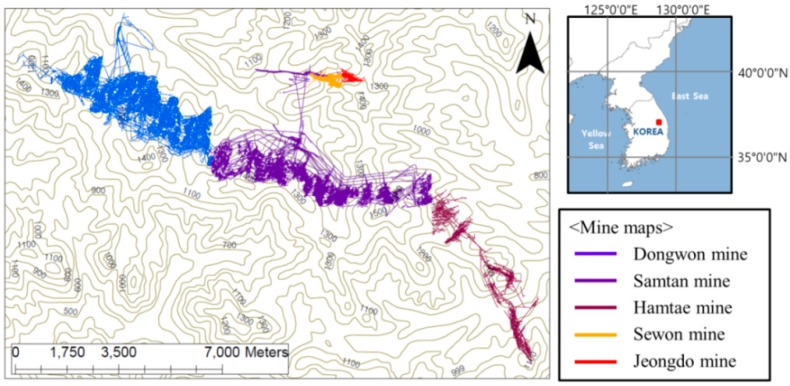
Mine maps of the Dongwon coal mine and surrounding mines.

A number of other mines were in operation near the Dongwon coal mine, such as the Samtan, Hamtae, Eoryong, Donghae, Jeongdong, Jangwon, Gyeongil, and Sewon mines, but all of these were abandoned prior to the closure of the Dongwon mine. The Coal Industry Promotion Board [23] analyzed the connectivity between the coal mines in the Gohan/Sabuk district, and described potential underground water movement between the Dongwon, Samtan, Sewon, Jeongdong, and Hamtae coal mines after abandonment, resulting from the connectivity between mining pits because of the mines’ geographical proximity. In this study, the Dongwon, Samtan, Sewon, Jeongdong, and Hamtae coal mining areas located in the Gohan/Sabuk district are used as study areas to test whether SIMPL can be applied to relatively extensive abandoned coal mine sites (Figure 8). The groundwater rebound phenomenon in the shaft of the Dongwon coal mine was analyzed; here, changes in the intra-pit groundwater level and water quality have been monitored since 2005, as reported by Cheong et al. [24].

For data input into the SIMPL program, the Dongwon coal mine was set as Pond 1, and the Samtan, Sewon, Jeongdong, and Hamtae coal mines were together set as Pond 2; information about the two ponds and a pipe connecting them was input into SIMPL, as shown in Table 3. The area of the catchment basin in the study area was calculated approximately by a hydrological analysis using a 150 m resolution Digital Elevation Model (DEM); Geographic Information System (GIS) methods for hydrological analysis of mining areas are described in detail by Choi [25], and Yi et al. [26]. The initial water level input for Pond 1 was 221.5 m above MSL, as measured in the field on 2 July 2005. The initial water level input for Pond 2 was 750 m above MSL, the groundwater level recorded by Samtan Jeongam Mining Company at the time the Dongwon coal mine was abandoned. The amount of daily rainfall (mm/d) and annual evaporation (1200 mm/yr) in the Taebaek district were input into the SIMPL program, with data provided by the Korea Meteorological Administration (KMA, Seoul, Korea), The simulation was carried out at a daily interval from 2 July 2005 (Day 0) to 31 January 2008 (Day 943). The pipe parameters and storage coefficients of Pond 1 and Pond 2 were corrected following comparison of the field monitoring data with the prediction results. Table 4 shows the correlation coefficient and RMSE between simulated and observed groundwater levels according to pipe parameter P that is proposed in SIMPL. Correlation coefficients are very high for all cases. Therefore, the value of 1.2 was determined as parameter P by the result of RMSE.

**Figure 9:**
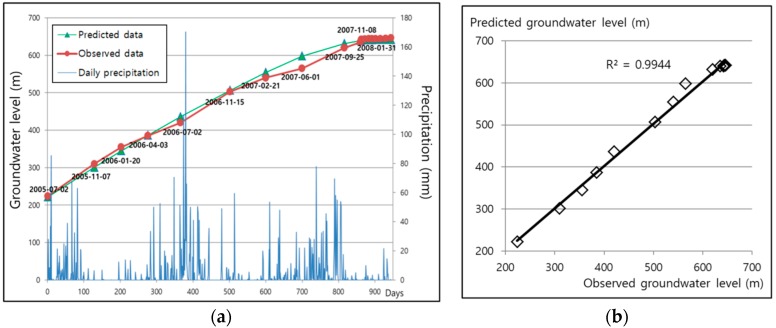
Results from the Dongwon coal mine. (**a**) Simulated and observed groundwater levels at the shaft; (**b**) Scatter plot of simulated and observed groundwater levels.

Figure 9 shows both the SIMPL simulation results and observational records of the groundwater level in the shaft of the Dongwon coal mine by using the value of 1.2 as pipe parameter P. The SIMPL simulation result was somewhat lower than the field monitoring result from July 2005 until April 2006, but was a little higher from July 2006 until November 2007; however, these differences were not large. After the groundwater level reached 639 m in November 2007, the increase in groundwater level slowed down, a result of groundwater flowing out to the surface at the 639 m point through cracks in the strata [24]; this is also seen in the field monitoring data. This Dongwon coal mine case study confirms that SIMPL can be successfully used for groundwater rebound prediction for extensive areas of abandoned mines.

### 4.2. Dalsung Copper Mine

The Dalsung copper mine is located in the Dalsung district, Daegu-si, and produced approximately 50,000 tons of copper and 2000 tons of tungsten until its closure in 1994. Copper and tungsten ore bodies are deposited as hydrothermal mineral deposits of width ~100 m and length ~200 m, with the vertical interval between the levels developed to be 20 m. Mining work was carried out from 250 m above MSL, at the earth’s surface (0 level), up to 310 m (upper 3rd level), and down to 60 m (lower 10th level). After the mine was abandoned, all pitheads were closed except that of the 0-level drift. Since the levels were well inter-connected, heavy rainfall in 1976 induced subsidence of the ground above the goafs. Although the ground was reclaimed in 1996, rainwater flowing into the goafs caused acid mine drainage.

**Figure 10:**
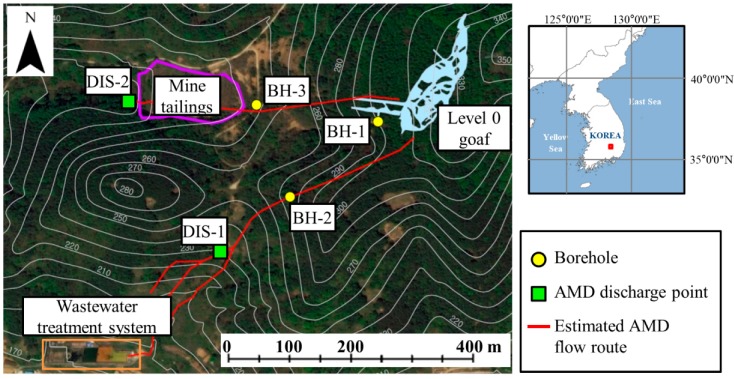
Mine maps of the Dalsung copper mine.

Outflows of acid mine drainage were observed at two sites, as shown in Figure 10. The first was the slope near the wastewater treatment system (DIS-1), and the second was the slope near the mine tailing dumps (DIS-2). The Mine Reclamation Corporation (MIRECO) [27] analyzed the mine connectivity using seismic surveys and described the possibility of underground water movement between the mine workings and two outflow sites (Figure 10). In this study, the area including the mine workings, wastewater treatment system, and mine tailing dumps was set as the study area in order to test whether SIMPL can be successfully applied to relatively local abandoned mine sites. The groundwater rebound phenomenon in the Dalsung copper mine was also analyzed; for the Dalsung copper mine, groundwater levels in the mine area (BH-1) and near the sites where acid mine drainage outflows (BH-2 and BH 3) have been monitored since measuring instruments were installed in 2016.

For data input into the SIMPL program, the mining area with drifts and goafs was set as Pond 1, and the two sites where acid mine drainage outflows were set as Pond 2 and Pond 3. The data about the three ponds and connecting pipes were input into the SIMPL program as shown in Table 5. The heights of the discharge points and connections between the ponds were determined based on the results of a boring investigation [27]. The area of the study area catchment basin was calculated approximately via a hydrological analysis using a 30 m resolution DEM. The initial water level inputs for Ponds 1, 2, and 3 were 243.0 m, 239.2 m, and 229.1 m above MSL, respectively, based on the observational data. The daily rainfall (mm/d) data and annual evaporation value (1200 mm/a) in the Daegu-si were input into the SIMPL program. The simulation was carried out on a daily interval from 2 May 2016 (Day 0) until 23 August 2017 (Day 478). Following comparison of the field monitoring data with the SIMPL prediction results, the storage coefficients were corrected to multiple values. Since Pond 1 was located near the underground voids generated by mining activities, its storage coefficients were relatively higher than those of the other ponds. Table 6 shows the correlation coefficient and RMSE between simulated and observed groundwater levels according to pipe parameter P. The value of 0.6 was determined as parameter P by considering RMSE and groundwater level variations although the highest correlation coefficient is calculated at the parameter P of 0.7.

**Figure 11:**
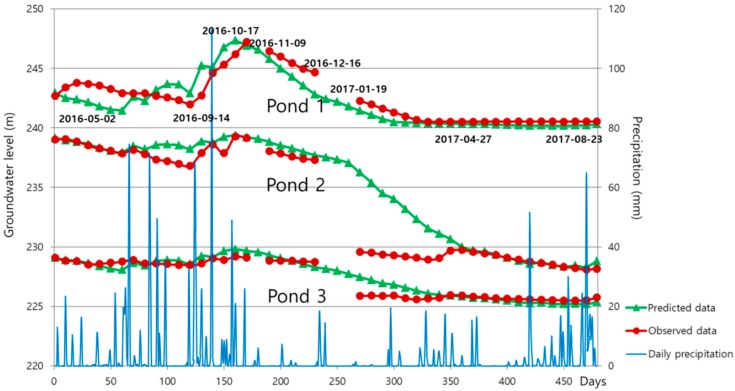
Simulated and observed groundwater levels at the three ponds in the Dalsung copper mine.

**Figure 12:**
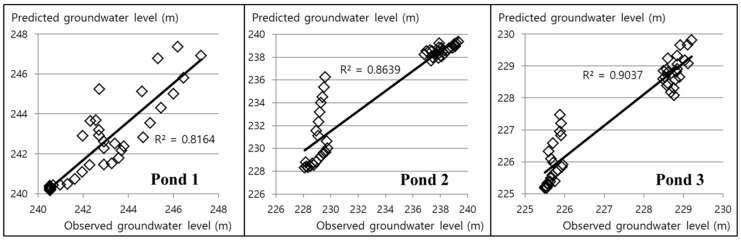
Scatter plots of simulated and observed groundwater levels at the three ponds in the Dalsung copper mine.

**Figure 13:**
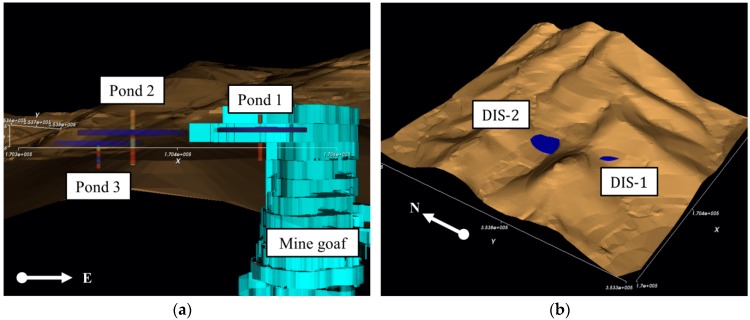
3D objects of the Dalsung copper mine constructed in the SIMPL program. (**a**) Ponds and mine goaf; (**b**) Groundwater flows at the discharge points.

Figure 11 shows the SIMPL simulation and field monitoring results of the groundwater level in the Dalsung copper mine by using the value of 0.6 as pipe parameter P. Observational records have a gap of 22 days from 18 October 2016 to 8 November 2016, and a gap of 33 days from 17 December 2016 to 18 January 2017, because of problems with the monitoring data. Simulation results for Pond 1 are a little lower than those determined by field monitoring from 19 January 2017 to 27 April 2017, and simulated results for Pond 2 and Pond 3 are somewhat higher from 19 January 2017 to 27 April 2017. These results are also shown on the scatter plots in Figure 12. However, the sign of the groundwater level variation was well predicted, and the quantitative difference was not large, considering the scale. In addition, these rapid drops of observed data may be the result of monitoring errors which caused gaps in the records. The groundwater level of Pond 1 changed only a small amount after the groundwater level dropped to 240 m above MSL because of the high storage coefficients of Pond 1, which is located near the underground voids. It can be seen in Figure 13a that the groundwater level of Pond 1 is similar to the height of the mine goaf. Although the groundwater levels of the three ponds decreased over the monitoring period, acid mine drainage was still a problem in this area. Because the groundwater levels of the three ponds were higher than 225 m for the period, acid mine drainage outflowed at the two discharge points with elevations of 210 m and 190 m. Figure 13b intuitively illustrates how groundwater flows out to the discharge points because the water levels are higher than the levels of the discharge points. To prevent outflow of acid mine drainage from the Dalsung copper mine, the groundwater level should be reduced to <190 m. Reclamation of upper ground subsidence may be one potential way to cause a drop in the groundwater level.

## 5. Discussion

The sizes of mine workings are very diverse, ranging in size from several hundred square meters (small mine workings) to several thousand square kilometers (locally connected mines). Moreover, the time scale for consideration of the groundwater rebound phenomenon of mine workings is diverse, ranging from several months (small mine workings) to several decades (locally connected mines). Accordingly, for predicting mine groundwater rebound, it is important to select a suitable groundwater flow model in accordance with the spatial scale of the mine workings and the temporal scale of the predicted time. This is because it is difficult for one type of groundwater flow model to provide reliable groundwater rebound prediction results for all the conditions of the spatial and temporal scales.

The MODFLOW model is based on the conventional Darcian groundwater flow equation for large systems, using the finite difference method to solve the groundwater equation. This type of groundwater flow model is primarily used when the largest spatial scale and the longest temporal scale should be considered. MINEDW is a model based on both Darcian and non-Darcian conditions, using a three-dimensional finite element groundwater flow code to solve the equation. It can be used to predict local and regional environmental impacts of mine dewatering and to simulate the infilling of a pit lake after mining ceases. Particularly, a finite element grid is useful for mining applications with complex geological structures and discharge points, such as drifts or drainholes. A simplified model, such as the GRAM model or the model proposed in this study, is a lumped parameter model that abstracts mine areas in the forms of ponds and pipes connected to each other. Although the model is not appropriate for modeling the groundwater distributions at a large spatial scale, it is designed to effectively predict the variation in the groundwater level according to time. Especially in the proposed model of this study, the limitations of the GRAM model were supplemented. The model in this study can consider inclined pipes and simplify parameters for the calibration process. In addition, 3D visualization abilities of the SIMPL program can help intuitively understand groundwater rebound and acid mine drainage outflows.

These three types of models can be utilized, stage by stage, to complement each other for predicting the mine groundwater rebound phenomenon in abandoned mine areas. The result of groundwater modeling at a large spatial scale by MODFLOW can help to define the geometric domain and boundary conditions for the specific groundwater modeling of MINEDW. In addition, the result of MINEDW groundwater modeling can serve as useful information for SIMPL to select suitable areas for monitoring and predicting the groundwater rebound phenomenon.

The SIMPL program developed in this study can be utilized in mine reclamation planning. If the groundwater rebound is less likely to occur as a result of the SIMPL program, the application of the acid mine drainage control method is less necessary. If the groundwater rebound problem is critical, on the other hand, the result of the SIMPL prediction can be used for a preliminary selection of an acid mine drainage control method. In addition, the application timing of an acid mine drainage control method can be estimated according to the rate of groundwater rebound. Therefore, the result is expected to be a useful indicator that can prioritize many mines and support decision-making during the planning of mine reclamation programs.

## 6. Conclusions

In this study, the SIMPL program was presented as a user-friendly 3D program created using Visual Basic 2013 and VTK to facilitate effective visualization of the results of groundwater level prediction analyses. In the simplified model, the data required for the modeling of the mine groundwater rebound phenomenon is relatively straightforward for the input, and the application of the model to abandoned mine areas is fairly simple. Most of the required input data and 3D objects can be generated by processing data, such as digital maps and drift maps, through GIS-based spatial analysis. To demonstrate a practical application of the software, the groundwater rebound phenomena in the Dongwon coal mine and Dalsung copper mine were simulated using the SIMPL program. Following comparison of the simulation results with field monitoring of the groundwater level data, changes in the simulated and observed groundwater levels were shown to be similar. The results demonstrate that SIMPL can be applied to abandoned mine areas in a range of spatial scales and environments.

While it is straightforward to apply SIMPL to the abandoned mine areas for which field survey data are difficult to obtain, we note that the simplified model has several limitations. First, it is difficult to accurately determine the storage coefficient of the mine at an actual mine site, so the groundwater rebound prediction result can vary greatly depending on the coefficient value that is input into the model. Second, the simplified model does not take into account groundwater supply sources, such as adjacent streams; these could influence the simulation results, because the model considers neither the groundwater that flows into the area from the streams nor the outflow of the groundwater to these streams. While many mines have artificial structures installed to prevent the inflow and outflow of groundwater from/to adjacent streams, this is not universal. Third, the simplified model assumes that there is no hydraulic gradient in a mine, but this is not always the case in real mines. This limitation could be compensated for by defining more ponds with monitoring instruments. It thus requires future improvement in several respects.

While there are some limitations to the simplified model, it is a remarkably powerful for simple and straightforward field applications considering groundwater rebound in the area surrounding a mine. The SIMPL program, which analyzes the groundwater level and visualizes the results as 3D objects, makes application of the groundwater rebound prediction more convenient and intuitive. The SIMPL program is, therefore, a useful new tool for practical applications, like mine reclamation programs. Since additional modules can be easily added to the SIMPL program, the software could be made even more applicable to abandoned mine areas following future work, with the addition of new modules incorporating, for example, geochemical modeling of acid mine drainage.

## Figures and Tables

**Table 1:** SIMPL program input and output data.

Type	Data
**Input**	Precipitation (mm) Evaporation (mm/year) Attenuation of recharge over time Area of each pond (m^2^) Area of surface catchment of each pond (m^2^) Number of pond connections and, for each, their height (m), diameter and parameter P ($\sqrt{\left(m/s^{2}\right)}$) Number of surface discharge points and, for each, their height (m), diameter and parameter P ($\sqrt{\left(m/s^{2}\right)}$) Storage coefficient Original water level (m) (Optional) Percentage runoff (%) (Optional) Abstractions (m^3^/timestep) (Optional) Marine inflow (m^3^/timestep) (Optional) Inflow from adjacent mines or aquifers (m^3^/timestep)
**Output**	Predicted groundwater height (m) of ponds

**Table 2:** Design of 3D objects in SIMPL.

Object	VTK Class Type	Data File Structure
Topographic map	vtkDelaunay2D	Line 1: 1 (Classification code) Line 2: Unused Line 3: Unused Line 4: (*x*, *y*, *z*) of point 1 Line 5: (*x*, *y*, *z*) of point 2 ...
Drift or shaft	vtkLineSource vtkTubeFilter	Line 1: 2 (Classification code) Line 2: Radius of tube Line 3: Unused Line 4: (*x*, *y*, *z*) of start point 1 / end point 1 Line 5: (*x*, *y*, *z*) of start point 2 / end point 2 ...
Borehole	vtkLineSource vtkTubeFilter	Line 1: 3 (Classification code) Line 2: Radius of tube Line 3: Number of class Line 4: (*x*, *y*, *z*) of point 1 / label / class Line 5: (*x*, *y*, *z*) of point 2 / label / class ...
Goaf	vtkPolygon vtkLinearExtrusionFilter	Line 1:4 (Classification code) Line 2: Extrusion length Line 3: Unused Line 4: (*x*, *y*, *z*) of point 1 / polygon number Line 5: (*x*, *y*, *z*) of point 2 / polygon number ...
Groundwater level	vtkCylinderSource	Line 1: 5 (Classification code) Line 2: Number of ponds Line 3: Radius of each pond Line 4: Number of time steps Line 5: Pond number / time step / (*x*, *y*, *z*) of water level ...

**Table 3:** Values input into the SIMPL program for prediction of the Dongwon coal mine groundwater rebound.

Entity	Parameters	Input Values	
**Pond**	Pond number	1	2
	Pond area	36 km^2^	150 km^2^
	Area of surface catchment	50 km^2^	200 km^2^
	Storage coefficient	0.45	0.45
	Original water level	221.5 m	750 m
	Level of surface discharge point	639 m (fractures)	-
**Pipe**	Connected ponds	Ponds 1 and 2	
	Height	570 m	
	Pipe diameter D	2 m	
	Pipe parameter P	1.2 $\sqrt{\left(m/s^{2}\right)}$	

**Table 4:** The result of correlation coefficients and RMSE between simulated and observed groundwater levels according to pipe parameter of the Dongwon coal mine.

Pipe Parameter P	Correlation Coefficient	RMSE
0.6	0.997	11.884
0.8	0.997	10.147
1.0	0.998	8.664
1.2	0.998	8.308
1.4	0.998	10.287
1.6	0.998	15.279

**Table 5:** Values input into the SIMPL program for prediction of the Dalsung copper mine groundwater rebound.

Entity	Parameters	Input Values			
**Pond**	Pond number	1	2		3
	Pond area	100,000 m^2^	100,000 m^2^		100,000 m^2^
	Area of surface catchment	2.5 km^2^	2 km^2^		2 km^2^
	Storage coefficient	0.3–1.0	0.1–0.3		0.3–0.7
	Original water level	243.0 m	239.2 m		229.1 m
	Level of surface discharge point	230.0 m (fractures)	210.0 m (fractures)		190.0 m (fractures)
**Pipe**	Connected ponds	Ponds 1 and 2		Ponds 2 and 3	
	Height	241.0 m		225.0 m	
	Pipe diameter D	0.05 m		0.05 m	
	Pipe parameter P	0.6 $\sqrt{\left(m/s^{2}\right)}$		0.6 $\sqrt{\left(m/s^{2}\right)}$	

**Table 6:** The result of correlation coefficients and RMSE between simulated and observed groundwater levels according to pipe parameter of the Dalsung copper mine.

Pipe Parameter P	Pond Number					
	1	2	3	1	2	3
	Correlation coefficient			RMSE		
0.3	0.850	0.918	0.881	1.635	2.228	1.328
0.4	0.855	0.923	0.909	1.365	2.147	0.935
0.5	0.889	0.924	0.937	1.056	2.105	0.650
0.6	0.911	0.934	0.957	0.901	1.921	0.469
0.7	0.919	0.945	0.966	1.022	1.714	0.537
0.8	0.857	0.955	0.964	2.056	1.493	0.793
